# Association of time in blood glucose range with outcomes following cardiac surgery

**DOI:** 10.1186/1471-2253-15-14

**Published:** 2015-01-26

**Authors:** Amr S Omar, Ahmed Salama, Mahmoud Allam, Yasser Elgohary, Shaban Mohammed, Alejandro Kohn Tuli, Rajvir Singh

**Affiliations:** Department of Cardiothoracic Surgery/Cardiac Anaesthesia & ICU Section, Heart Hospital, Hamad Medical Corporation, Doha, PO: 3050, Qatar; Department of Critical Care Medicine, Beni Suef University, Beni Suef, Egypt; Department of Anesthesia, Al-Azhar University, Cairo, Egypt; Department of Clinical Pharmacy, Hamad Medical Corporation, Doha, Qatar; Department of Medial Research, Hamad Medical Corporation, Doha, Qatar

**Keywords:** Glucose control, Outcome, Wound infection

## Abstract

**Background:**

The importance of optimal postoperative glycemic control in cardiac patients remains unclear. Various glycemic targets have been prescribed to reduce wound infection and overall mortality rates.

Aim of the work: To assess glucose control, as determined by time in range (TIR), in patients with glycemic targets of 6.0 to 8.1 mmol/L, and to determine factors related to poor control.

**Methods:**

This prospective descriptive study evaluated 227 consecutive patients, 100 with and 127 without diabetes, after cardiac surgery. Patients received insulin to target glucose concentrations of 6.0 to 8.1 mmol/L. Data analyzed included patient age, gender, race, Euro score, cardiopulmonary bypass time (CPB), aortic cross clamp time (ACC), length of ventilation, stay in the intensive care unit (ICU) and stay in the hospital. Patients were divided into two groups, those who maintained > 80% and < 80% TIR. Outcome variables were compared in diabetics and non-diabetics.

**Results:**

Patients with >80% and <80% TIR were matched in age, sex, gender, and Euro score. Failure to maintain target glycemia was significantly more frequent in diabetics (p = 0.001), in patients with glycated hemoglobin (HbA1c) > 8% (p = 0.0001), and in patients taking dopamine (p = 0.04) and adrenaline (p = 0.05). Times of CPB and ACC, length of stay in the ICU and ventilation were significantly higher in patients with TIR <80% than >80%. Rates of hypoglycemia, acute kidney injury, and in-hospital mortality were similar in the two groups, although the incidence of wound infection was higher in patients with TIR <80%. Both diabetics and non-diabetics with low TIR had poorer outcomes, as shown by length of stay and POAF. No significant differences were found between the two ethnic groups (Arabs and Asians).

**Conclusion:**

Patients with >80% TIR, whether or not diabetics, had better outcomes than those with <80% TIR, as determined by wound infection, lengths of ventilation and ICU stay. Additionally, they were not subject to frequent hypoglycemic events. Preoperatively high HbA1C is likely a good predictor of poor glycemic control.

## Background

Tracking adherence to insulin infusion protocols and the use of standard metrics are key to maintaining glucose control. Control practices after cardiac surgery were shown to reduce mediastinitis [1]. Poor perioperative control of blood glucose concentration may be associated with poorer outcomes in patients undergoing cardiac surgery [2].

Tight glucose control has been reported to improve acute outcomes in hospitalized diabetic patients, including risks of infection and death [3, 4]. Tight glucose control, through continuous intravenous injection of insulin, was also found to reduce mediastinitis and mortality rates, as well as costs and length of hospital stay, in diabetic patients after cardiac surgery [2–5]. Other advantages of tight glucose control in cardiac surgery patients include reductions in the rates of postoperative atrial fibrillation (POAF) and ischemia [5].

Although maintaining normoglycemia between 4.4 and 6.1 mmol/L using intensive insulin therapy reduced mortality in the surgical intensive care unit (ICU), as well as organ complications associated with critical illness, tight insulin control was also associated with frequent hypoglycemic episodes [6]. Moreover, a recent study showed that intensive insulin therapy (6.1–8.3 mmol/L) yielded superior outcomes when compared with less intense control targeting higher glucose concentrations [7].

Cardiopulmonary bypass (CPB) has been shown to affect glucose control, with controlling blood glucose being difficult during cardiac surgery [8]. Tight blood glucose control during CPB was not easily achieved owing to the exacerbation of insulin resistance. In addition [9], an investigation of blood glucose homeostasis derangement showed that glucose levels were increased and insulin levels decreased during hypothermic CPB [9].

Insulin resistance has been associated with increased levels of inflammatory cytokines in critically ill patients. Concomitant insulin resistance plus hypothermia may be aggravated by insulin adherence to the plastic material included in the extracorporeal circuit, by glucose administration in cardioplegia solution, and by the steroids that may be used to reduce inflammatory responses to CPB. Blood glucose concentrations tend to increase after rewarming, as do levels of catecholamines, glucagon, and growth hormone [10]. The underlying molecular mechanisms of this insulin resistance are not fully understood, although transmembrane protein defects are thought to play a role [11].

Ethnic differences in insulin sensitivity have been associated with variations in body fat. South Asian adolescents are more insulin resistant, with more body fat, than white European adolescents, which may contribute to the higher risk in the former of developing type 2 diabetes [12]. Detrimental effects of hyperglycemia may affect critically ill non-diabetic and diabetic patients. The harmful effects of intensive hyperglycemic control are still considered speculative [13], and the three domains of glycemic control hyperglycemia, hypoglycemia and glucose variability could affect outcomes in critically ill patients [14]. This study therefore evaluated patient outcomes after implementation of a validated insulin protocol [15], by assessing time in range (TIR) to investigate factors related to poor glucose control.

## Methods

This prospective, descriptive, single-center study with purposive sampling evaluated 227 consecutive patients, 100 with and 127 without diabetes, after cardiac surgery. Patients who received insulin for at least 12 h were eligible. The study was performed from September 2012 to August 2013 in the 12-bed cardiothoracic ICU of Hamad Medical Corporation. The study was approved by the ethics committee of Hamad Medical Corporation (reference number 13156/13), which waived the requirement for informed consent, since no specific intervention was performed and blood sampling was part of routine care to control blood glucose post-operatively.

Mean blood glucose (BG) concentration was measured during infusion of 1.0 unit/mL of insulin at a rate sufficient to maintain a target glucose concentration of 6.0–8.1 mmol/L. Arterial blood was sampled every 1 h during the first 6–12 h after surgery, with capillary blood samples obtained by finger stick thereafter. BG concentrations were measured using the Accu-Check Inform II point-of-care meters (Roche Diagnostics, Indianapolis, IN). A quality control program was maintained to assess nurses’ compliance with and interpretation of the protocol. Nurses recorded BG concentrations, measurement times and insulin infusion rates on a daily ICU chart.

The main objective of this study was to investigate glucose control, using TIR 80% as the threshold, and factors associated with poor control. Total time of insulin infusion (A) and the period of time being within the target range (B) were measured in each patient during insulin infusion, with TIR calculated as B/A*100. Patients were divided into two groups based on successful maintenance of TIR, with Groups I and II consisting of individuals with TIRs >80% and <80%, respectively. Hypoglycemia was defined as BG <4 mmol/L and severe hypoglycemia as BG <2.2 mmol/L.

Factors assessed at admission to the ICU included age, sex, race, medical diseases, drugs, type of surgery, anesthesia time, CPB time, aortic cross clamp (ACC) time, use of inotropes and vasopressors, Euro SCORE, statin therapy, length of mechanical ventilation, and stay in the ICU and the hospital. Complications and outcomes, including acute kidney injury (AKI), POAF, infection, stroke, wound infection, and death, were recorded for each patient. Data were retrieved using Dendrite Clinical Systems (London, UK). Outcomes were compared in subgroups of diabetics and non-diabetics, and factors associated with poor glycemic control were analyzed. ICU stay was dichotomized as ≤48 hours and >48 hours.

### Statistical analysis

Normally distributed continuous variables are reported as mean ± SD, non-normally distributed continuous variables as median and range, and categorical variables as frequency and percentage. Normally and non-normally distributed continuous variables were compared using Student’s t-tests and Mann–Whitney U tests, respectively, and categorical variables using Chi squared tests. A two-sided P-value <0.05 was considered statistically significant. Variables influencing TIR in our and previous analyses were assessed by multivariate regression analysis. All statistical analyses were performed using SPSS Version 16 software (SPSS Inc. Chicago, IL, USA).

## Results

Of the 260 patients screened, 227 were enrolled; the remaining 33 patients were excluded because they were infused with insulin for < 12 h. The study population consisted mostly of males and had a mean age of 54.3 ± 10.8 years (Table 1); in addition, 43.1% of the patients were diabetics, and 59.3% were hypertensive. There was a higher proportion of Asians than Arabs in the studied population. The majority of patients underwent CABG surgery (Tables 1 and 2).

**Table 1:** **Demographic and clinical characteristics of the included patients**

Variable	Number	Minimum	Maximum	Mean ± SD
**Age**	227	15	78	54.3 ± 10.8
**BMI** (kg/m^2^)	226	14.5	44.8	27.4 ± 5.1
**Creatinine** (micromole/L)	221	14.4	746	92.4 ± 53.1
**EF%**	226	22	65	49 ± 9.6
**HgA1C (%)**	205	5	66	7.4 ± 4.5
**Additive Euro score**	222	0	17	3.6 ± 2.9
**CPB time** (minutes)	210	0	304	110.2 ± 46.3
**ACC time** (minutes)	206	0	164	71.6 ± 35.8
**WBCs, × 10** ^**6**^ **/ml**	214	5	30	12.1 ± 4.4
**Hb, g/dl**	217	7	16	10.2 ± 1.5
**Anesthesia time** (minutes)	227	180	700	323 ± 90
**LOV** (minutes)	227	180	4800	532 ± 501
**LOS**_**hosp**_ (days)	226	4	499	31.7 ± 29.9

BMI, body mass index; EF, ejection fraction; HgA1C, glycated hemoglobin; CPB, cardiopulmonary bypass; ACC, aortic cross clamp; WBCs, white blood cells; LOV, length of mechanical ventilation, LOS_hosp_, length of hospital stay.

**Table 2:** **Comparative characteristics of the included patients**

Variable	Number (%)
**Gender**	
Male	205 (90.3)
Female	22 (9.7)
***Hypertension***	135 (59.5)
***Diabetes***	
*Type I*	34 (14.9)
*Type II*	66 (29)
**Diabetes treatment**	
*None*	126 (55)
*Oral hypoglycemic*	75 (33)
*Insulin*	25 (11)
**Ethnicity**	
*Qatari*	12 (5.3)
*Arab*	75 (33)
*Asian/Others*	140 (61.7)
**Smoking**	
*Never smoker*	88 (38.8)
*Ex-smoker*	101 (44.5)
*Current smoker*	30 (13.2)
**Surgery type**	
*CABG*	167 (73.6)
*Valvular surgery*	49 (21.6)
*Adult congenital*	5 (2.2)
*Aortic dissection*	5 (2.2)

Patients were divided into two groups based on their success in maintaining target BG concentration. Group I consisted of patients with TIR >80% and Group II of patients with TIR < 80% (Table 3). The two groups were well matched in age, gender, BMI, association with hypertension, and Euro score. Non-diabetics showed better BG control than diabetics (Table 3). There were no significant differences between ethnic groups. Basal creatinine and EF% were similar in Groups I and II. HbA1C was significantly higher in Group II. Patients taking dopamine and adrenaline had poorer BG control, as were patients who underwent CABG. In contrast, patients who underwent valvular surgeries were more likely to have better BG control.

**Table 3:** **Clinical and laboratory variables of patients in Groups I (TIR >80%) and II (TIR <80%)**

Variable	Group I N = 146 (%)	Group II (N = 81)	P-value
**Age**	54.1 ± 11	54.6 ± 11	0.08
**Sex male**	135 (92.5)	70 (86.4)	0.1
**Hypertension**	86 (58.9)	49 (60.4)	0.15
**Non diabetics**	99(68.3)	27 (33.3)	0.01
**IDDM**	36 (24.8)	39 (48.1)	0.001
**NIDDM**	10 (6.9)	15 (18.5)	0.001
**BMI**	27.4 ± 4.8	28.7 ± 5.9	0.4
**Ethnicity**			
**Arab**	57 (39)	30 (37)	0.13
**Asian**	89 (61)	51 (63)	0.3
**Euro score**	3.5 ± 2.5	3.8 ± 3.5	0.47
**Basal creatinine (micromole/L)**	88.6 ± 25.3	94.7 ± 63	0.8
**EF%**	49.7 ± 8.6	48.7 ± 10.1	0.4
**HbA1c %**	6.6 ± 1.7	8 ± 2.2	0.001
**Surgery (elective)**	97 (66.4)	57 (70.4)	0.35
**Inotrops**			
**Dopamine**	20 (13.8)	19 (24.7)	0.03
**Adrenaline**	16 (11.1)	18 (23)	0.04
**Noradrenline**	40 (27.4)	17 (21)	0.1
**Surgery**			
**CABG**	100 (68.5)	67 (83.3)	0.04
**Valvular**	40 (27.5)	9 (11.3)	0.03
**Aortic disssection**	3 (2.1)	2 (2.5)	0.1
**Adult congenital**	3 (2.1)	2 (2.5)	0.1

IDDM, insulin dependent diabetes mellitus; NIDDM, non insulin dependent diabetes mellitus; BMI, body mass index; HbA1C, glycated hemoglobin; EF, ejection fraction; CABG, coronary artery bypass graft.

Table 4 summarizes the clinical outcomes in these patients. ACC and total anesthesia time were significantly higher in Group II, as were lengths of ICU and hospital stay and duration of mechanical ventilation. The percentages of patients with new POAF and wound infection were significantly higher in Group II, as was the rate of overall in-hospital mortality. Multivariate analysis showed that diabetes was the only independent predicator of poor glycemic control (Table 5). Variables affecting TIR were included in the multivariate model (Table 6). Comparisons of outcome variables in subgroups of diabetics and non-diabetics showed that TIR <80% was associated with longer lengths of stay and higher POAF frequency in both diabetics and non-diabetics (Tables 7 and 8). Moreover, multivariate analysis showed that TIR <80% was significantly associated with longer ICU stay (Table 9).

**Table 4:** **Clinical outcomes of patients in Groups I (TIR >80%) and II (TIR <80%)**

Variable	Group I (N = 146)	Group II (N = 81)	P-value
**Intraoperative parameters**			
**CPB time (minutes)**	107.6 ± 47	112.7 ± 45	0.4
**ACC time (minutes)**	64.8 ± 37	75.5 ± 23	0.04
**Anesthesia time (minutes)**	318 ± 103	349 ± 81	0.05
**Postoperative parameters**			
**LOS**_**ICU**_ **median (range) (hours)**	203 ± 142 (83–540)	256 ± 411 (46–2140)	0.04
**LOS**_**hosp**_ **median (range) (days)**	7.5 ± 3.7 (3.6-22)	9.9 ± 11 (3.9-73)	0.03
**LOV median (range) (minutes)**	455 ± 233 (200–1440)	574 ± 597 (180–4800)	0.03
**Postoperative complications**			
**Wound infection**	3 (2.1)	7 (8.6)	0.05
**AF**	10 (6.8)	12 (14.8)	0.04
**AKI**	7 (4.9)	7 (8.9)	0.17
**VAP**	0	1 (1.2)	
**Hypoglycemia**	2 (1.3)	2 (2.4)	
**Early stroke**	0	1 (1.2)	
**Inhospital mortality**	2 (1.3)	3 (3.7)	

CPB, cardiopulmonary bypass; ACC, aortic cross clamp; LOV, length of mechanical ventilation; LOS_ICU_, length of ICU stay; LOS_hosp_, length of hospital stay; AF, atrial fibrillation; AKI, acute kidney injury; VAP ventilator associated pneumonia.

**Table 5:** **Multivariate logistic regression analysis for low TIR less than 80%**

Variable	Adjusted OR	95% C.I.	Significance
**Age**	0.99	0.95 – 1.03	0.68
**Gender female**	0.29	0.07 – 1.21	0.09
**DM**	0.30	0.12 – 0.75	0.01
**Inotrops**	2.02	0.94-4.35	0.07
**Surgery**			
CABG	0.72	0.04-13.6	0.83
Valvular	1.34	0.73-24.7	0.84
**HbA1c**	0.8	0.66-1.02	0.07
**LOV** (minuts)	1	0.9-1.001	0.4
**LOS**_**ICU**_ (hours)	.99	0.9-1.006	0.6
**LOS**_**Hosp**_ **(days)**	.99	0.91-1.07	0.8
**ACC time**	1.01	0.9-1.06	0.3
**AF**	0.37	0.12-1.15	0.08

CABG coronary artery bypass graft, HbA1C glycated hemoglobin, LOV length of mechanical ventilation, LOS_ICU_ ICU length of stay, LOS_hosp_ Hospital length of stay, ACC aortic cross clamp time, AF atrial fibrillation.

**Table 6:** **Glycemic changes in Groups I (TIR >80%) and II (TIR <80%)**

Variable	Group I (N = 146)	Group II (N = 81)	P-value
**Normoglycemia**	35.1 ± 35.9	25.4 ± 10.9	0.003
**Hyperglycemia**	3.2 ± 5.3	10.2 ± 5.9	0.000
**Compliance**	34.1 ± 34.8	26.8 ± 11.5	0.025
**Total hours of insulin infusion**	37.7 ± 38	36 ± 15	0.62

**Table 7:** **Outcome variables in non-diabetics in both groups**

Variable	Group I (Non-diabetics) N = 99	Group II (Non diabetics) N = 27	P-value
**Complications**			
AKI	6 (6)	4 (14.8)	0.11
Nosocomial infections	3 (3)	0	
Inotrpope need	55 (55.1)	17 (62.9)	0.14
AF	10 (10.1)	6 (22.2)	0.05
**Postoperative parameters**			
**LOS**_**ICU**_ median (hours)	199 ± 143	244 ± 311	0.05
**LOS**_**hosp**_ median (days)	7.6 ± 4.7	9.3 ± 11	0.05
**LOV** median (minutes)	481 ± 291	588 ± 675	0.04

LOV length of mechanical ventilation, LOS_ICU_ ICU length of stay, LOS_hosp_ Hospital length of stay, AF atrial fibrillation, AKI acute kidney injury.

**Table 8:** **Outcome variables in diabetics in Groups I (TIR >80%) and II (TIR <80%)**

Variable	Group I (dkabetics) N = 46 (%)	Group II (dkabetics) (N = 54)	P-value
**Complications**			
**AKI**	1 (2.2)	3 (5.6)	0.11
**Nosocomial infections**	2 (4.3)	7 (13)	0.09
**Inotrope need**	22 (47.8)	37 (64.9)	0.08
**AF**	1 (2.2)	5 (9.3)	0.01
**Postoperative parameters**			
**LOS**_**ICU**_ **median (hours)**	223 ± 154	269 ± 445	0.05
**LOS**_**hosp**_ **median (days)**	8.5 ± 3.7	10.6 ± 11	0.03
**LOV median (minutes)**	442 ± 198	553 ± 452	0.01

LOV, length of mechanical ventilation; LOS_ICU_, length of ICU stay; LOS_hosp_, length of hospital stay; AF, atrial fibrillation; AKI, acute kidney injury.

**Table 9:** **Multivariate analysis for favorable ICU length of stay (=or < 48 hours)**

	Adjusted OR	95% CI	Significance
**Age**	1.000	0.971-1.031	0.990
**Gender**	0.976	0.313-3.039	0.966
**Diabetes**	0.811	0.387-1.701	0.580
**Inotrope needs**	2.169	1.147-4.102	0.017
**Surgery type**			
CABG	0.577	0.154-3.467	.692
Valvular	1.008	0.117-2.837	0.499
**ACC time**	2.240	0.998-1.017	0.101
**AF**	0.321	0.911-5.509	0.079
**TIR less than 80%**	0.668	0.159-.647	0.001

CABG coronary artery bypass graft, ACC aortic cross clamp time, AF atrial fibrillation, TIR time in range.

## Discussion

The main findings of this study were that patients with higher TIR had better postoperative outcomes, whether they were non-diabetics or diabetics, and that a BG range of 6.0–8.1 mmol/L was safe in the studied population. In addition, HbA1C was found to predict poor glycemic control, ethnicity was unrelated to poor control and hourly sampling of BG after cardiac surgery is useful in patient monitoring.

Adverse outcomes in surgical patients may be due to hyperglycemia [16, 17], and insulin therapy in the ICU has yielded diverse outcomes [6, 18]. Based on mortality, morbidity, and long-term survival benefits, the guidelines of the Society of Thoracic Surgeons recommended a glycemic control target of 6.1–8.0 mmol/L glucose after cardiac surgery [19]. More recently, moderate glycemic control after cardiac surgery showed better outcomes [20], and the literature relating to intensity of BG control in CV surgery patients is somewhat conflicted [21].

Diabetics comprised 43.1% of the patients in our study, compared with 13% and 20% in the Leuven and NICE-SUGAR studies, respectively. Diabetes is highly prevalent in populations, along with greater insulin resistance [22]. Using TIR to classify patients, we found that maintaining target TIR was more difficult in diabetics than in non-diabetics, confirming previous results showing that out of range glycemia was more prevalent in diabetics [23]. Although we hypothesized that poor control would be related to ethnicity, we found that both Asians and Arabs were equally distributed in both TIR groups.

Our finding, that use of dopamine or adrenaline was associated with poor glycemic control, is in good agreement with findings showing that higher BG levels were associated with adrenaline use [5]. Intensive glycemic management may be required in non-diabetic patients infused with high doses of catecholamines [24]. However, catecholamine infusion may be responsible for hyperglycemia in ICU patients [25].

We found that the frequency of poor glycemic control was higher in patients who underwent CABG than in those who underwent valvular surgery, a finding that may be due to the higher preoperative rate of diabetes in CABG patients. CABG was found to be more closely associated with hyperglycemia than was valvular surgery, as were wound infections (93% versus 4%) [3]. We found, however, that the acute nature of surgery was similar in both groups.

### Prediction of poor glycemic control

Although potential predictors of poor glycemic control, such as baseline creatinine concentration and EF%, were similar in our patient groups, HbA1C concentration was significantly higher in Group II. High HbA1c after CABG may be associated with higher short- and long-term mortality rates [26].

Hyperglycemia associated with CPB may be due to the insulin resistance that accompanies surgical intervention, resulting in poorer patient outcomes [27]. Although we found that CPB time was higher in Group II, the difference was not statistically significant. In contrast, ACC and total anesthesia time were significantly higher in Group II. Although insulin secretion is not impaired during cardiac surgery, insulin signaling cascade in target organs is reduced, and inotropes needed during weaning from CPB affect glucose levels [27]. CPB has been shown to affect glucose control [8], with increases in inflammatory cytokines during cardiac surgery enhancing insulin resistance [10].

### Postoperative parameters and complications

Lengths of stay in the ICU and hospital, as well as duration of mechanical ventilation, were all significantly higher in Group II, as well as in both non-diabetics and diabetics with low TIR. These results were consistent with findings showing that moderate glycemic control (6.6–10.0 mmol/L) in diabetic CABG patients was associated with minimal morbidity and mortality [28]. Moderate glycemic control (6.1–8.0 mmol/L) after cardiac surgery was found to reduce the duration of ventilation [29]. A 3-mmol/L increase in BG was found to be an independent predictor of deep sternal wound infection, length of stay in the hospital and mortality rate. In addition, new POAF events, blood transfusion and low cardiac output syndrome were found to correlate significantly [30], suggesting that reducing BG below 10 mmol/L appears to be an ideal goal. Maintaining BG below this concentration was associated with reductions in mortality and morbidity, whereas aggressive glycemic control (4.4–6.1 mmol/L) did not offer a superior advantage [31].

Complications related to poor glycemic control are challenging for health care practitioners. For example, we found that the occurrence of acute kidney injury (AKI) tended to be higher in Group II, similar to results showing that glycemic control after cardiac surgery was significantly associated with a reduced risk of AKI [32]. The rate of POAF events was also significantly higher in Group II, similar to findings showing that proper glycemic control could reduce the incidence of POAF after CABG, from 30% to 18% (39% risk reduction; p = 0.042) [29], and may reduce the rate of POAF-associated mortality [33]. In addition, a prospective randomized study found no differences in the rates of POAF events and wound infection between aggressive (5.0–6.6 mmol/L) and moderate (6.6–10.0 mmol/L) glycemic control [34]. We found that the rate of wound infection was significantly higher in Group II, similar to findings showing that the rate of wound infection was reduced from 2.6% to 1.0% following glycemic control for 18 months [35].

Wound infections after cardiac surgery may be reduced by antimicrobial prophylaxis, control of preoperative BG concentration, and staple avoidance in patients with a normal BMI [36]. Preoperative screening for diabetes may reduce the rates of these postoperative morbidities associated with surgical site infections [3]. We found that the rates of hypoglycemic events were similarly low in both groups, with two patients in each group experiencing hypoglycemia.

The overall in-hospital mortality rate was higher in Group II than in Group I (3.7% versus 1.2%). Outcomes may be improved by enhancing TIR in ICU settings, especially when hypoglycemia can be avoided. We found that both diabetics and non-diabetics with low TIR had similar outcomes, suggesting that diabetics with greater numbers of comorbidities may have poorer outcomes. Moreover, diabetics may benefit from higher target glucose concentrations [14].

### Strengths and limitations

Utilization of the TIR as a distinguishing factor may have clinical advantages. The relatively low rate of hypoglycemia in our study may have been due to the frequency of blood sampling (hourly during the first 6 h). This high sampling rate should not confer an extra burden on the nursing staff, as arterial sampling is required for early assessment of these patients. The optimum sampling frequency has not yet been determined, although sampling every 1–2 h is common in many studies [15, 37]. The early postoperative period is usually associated with stress from the use of inotropes, as well as bleeding, predisposing to early difficulties in glucose control. This study was limited by being performed at a single center, as well as by an inability to occlude the glucose variability.

## Conclusion

Patients with >80% TIR 6.0-8.1 mmol/L, whether diabetics or non-diabetics, had better outcomes than those with <80% TIR 6.0-8.1 mmol/L. The former group had a lower rate of wound infection, shorter duration of ventilation and shorter stay in the ICU. Moreover, strict glycemic control did not increase the occurrence of hypoglycemic events. Preoperatively high HbA1C appears a more likely predictor of poor glycemic control. Ethnicity had no effect on glucose control.

Recommendations and future directions

1. Attempt to enhance TIR in ICU population
2. HbA1C screening for all patients before cardiac surgeries.
3. Hourly sampling of blood glucose
4. Considering glucose variability in similar studies

Key messages

1. Power of TIR to predict outcome after cardiac surgeries.
2. Safety of 6.0-8.1 mmol/L target BG.
3. Reduced complication in the adequately controlled group
4. Power of HbA1C to predict poor control
5. Ethnicity is not predictive of poor control among the studied population
6. Value of hourly sampling of BG soon after cardiac surgery

## Consent

The ethics review panel waived informed consent for all patients enrolled in the study. However, all study data were maintained anonymously.

## Abbreviations

ACC: Aortic cross clamp
AKI: Acute kidney injury
BG: Blood glucose
CABG: Coronary artery bypass graft
CAD: Coronary artery disease
CPB: Cardiopulmonary bypass
HbA1c: Glycated hemoglobin
POAF: Postoperative atrial fibrillation
TIR: Time in range.
